# Molecular mechanism and potential therapeutic targets of liver metastasis from gastric cancer

**DOI:** 10.3389/fonc.2022.1000807

**Published:** 2022-11-09

**Authors:** Difeng Li, Xin Zhang, Lili Jiang

**Affiliations:** ^1^ The Sixth Affiliated Hospital of Guangzhou Medical University, Qingyuan People’s Hospital, Guangzhou Municipal and Guangdong Provincial Key Laboratory of Protein Modification and Degradation, School of Basic Medical Science, Guangzhou Medical University, Guangzhou, China; ^2^ Clinical Experimental Center, Jiangmen Key Laboratory of Clinical Biobanks and Translational Research, Jiangmen Central Hospital, Jiangmen, China; ^3^ Dongguan Key Laboratory of Medical Bioactive Molecular Developmental and Translational Research, Guangdong Provincial Key Laboratory of Medical Molecular Diagnostics, Guangdong Medical University, Dongguan, China; ^4^ Collaborative Innovation Center for Antitumor Active Substance Research and Development, Guangdong Medical University, Zhanjiang, Guangdong, China

**Keywords:** gastric cancer, liver metastasis, tumor microenvironment, therapeutic targets, molecular mechanism

## Abstract

Gastric cancer (GC) is characterized by high invasion and poor prognosis. The occurrence of liver metastasis seriously affects advanced GC prognosis. In recent years, great progress has been made in the field of GC liver metastasis. The abnormal expression of related genes leads to the occurrence of GC liver metastasis through metastasis cascades. The changes in the liver microenvironment provide a pre-metastasis condition for GC cells to colonize and grow. The development of several potential therapeutic targets might provide new therapeutic strategies for its treatment. Therefore, we reviewed the regulatory mechanism of abnormal genes mediating liver metastasis, the effect of liver resident cells on liver metastasis, and potential therapeutic targets, hoping to provide a novel therapeutic option to improve the quality of life and prognosis of GC patients with liver metastasis.

## Introduction

Gastric cancer (GC) is one of the most malignant tumors in the digestive tract and the fourth leading cause of cancer-related mortality worldwide. There were approximately 1.03 million new cases and 0.79 million deaths of GC in 2020 (1). In recent years, with the advancement of medical technology and health awareness, the incidence of GC has been decreasing (2, 3). GC develops due to multiple factors, *Helicobacter pylori* infection is considered a key pathogenic factor in GC development (4), while smoking, alcohol, poor diet, age, gender, and race are also potential risk factors (5). The incidence of GC can be reduced through appropriate management and control of risk factors. Clinically, it can be divided into two subtypes according to the criteria of Lauren classification: intestinal type and diffuse type. In clinical studies, Lauren classification can be used as an independent prognostic factor for GC patients with gastrectomy, and the prognosis of intestinal type is significantly better than that of diffuse-type (6, 7).

The tumor microenvironment (TME) is a complex and dynamic process in which tumor cells migrate, grow, metastasize and interact with resident cells. Tumor cells can change and maintain their conditions of survival and progression through autocrine and paracrine signaling to promote their growth and invasion (8). TME is composed of various elements, containing tumor cells, T-cells, B-cells, dendritic cells, myeloid-derived suppressor cells (MDSCs), tumor-associated macrophages (TAMs), carcinoma-associated fibroblasts (CAFs), tumor vasculature and lymphatics, and cytokines (9, 10). Although TME is the main mediator of tumorigenesis and growth, it is also an important site where novel tumor therapeutic regimens can come into play. By targeting a biological component or process in TME, tumor progression may be inhibited (11).

GC cells can reach solid organs throughout the body with blood flow. The unique structure and hemodynamic characteristics of the liver create favorable conditions for adherence and growth of tumor cells (12). As the largest glandular organ of the body, the liver is responsible for biosynthesis, host body defense, and substance metabolism and is supported by hepatic portal veins and hepatic arteries. Furthermore, paracrine signal transduction between liver resident cells and tumor cells further enhances the proliferation and invasion of tumor cells. Therefore, the purpose of this review was to elucidate the regulatory mechanism of GC liver metastasis and the effect of the liver microenvironment on tumor cells and find potential therapeutic targets for its treatment.

## Clinical diagnosis and treatment of liver metastasis from GC

As specific symptoms and biomarkers for early GC are not usually present, a large number of patients (approximately 70%) are in the advanced stage when they are diagnosed clinically, missing the optimal treatment period (13). Because of high invasion and poor prognosis, the median survival time of the advanced GC is < 12 months (14). Compared with advanced GC, the five-year survival rate of early GC after radical surgery is > 90%, and the prognosis is significantly better than that of advanced GC (15). The distant organ metastasis of GC usually develops through the blood channel, by which GC cells transfer to the liver, lung, brain, bone, and other organs. According to the epidemiological database survey, approximately 34% of GC patients suffer from distant metastasis, which is also one of the most common death causes of GC (16). The incidence of liver metastasis is higher than that of other distant organs (17).

The symptoms of GC liver metastasis are not obvious, including hepatomegaly, liver function abnormalities, jaundice, and ascites in severe cases. The presence of liver metastasis is often clinically observed by magnetic resonance imaging (MRI), computed tomography (CT), and positron emission tomography (PET) scan with an 18-fluorodeoxyglucose (FDG)-based tracer (18). CT is used widely, but MRI and PET have the advantage of detecting smaller lesions (19). In contrast, the identification of primary hepatocellular carcinoma and GC liver metastasis relies on the application of liver puncture biopsy. Palliative gastrectomy with synchronous liver metastasectomy is most frequently chosen and has the best prognostic modality (20). However transarterial therapy, chemotherapy, radiofrequency ablation, and immunotherapy provide effective alternatives for patients who cannot accept surgical resection (16). Therefore, it is important to reasonably assess the pathological characteristics and select the most appropriate treatment option for each patient individually.

Due to the high incidence of liver metastasis in patients with advanced GC, the prognosis is poor, and the five-year survival rate is < 10% (21). Therefore, liver metastasis is one of the most serious clinical problems in the treatment of advanced GC. Understanding the mechanisms of liver metastasis will help develop new treatment strategies, by which the survival rate of advanced GC can be improved.

## Biomolecules promote the occurrence of pre-metastasis behaviors by GC cells

**Figure 1:**
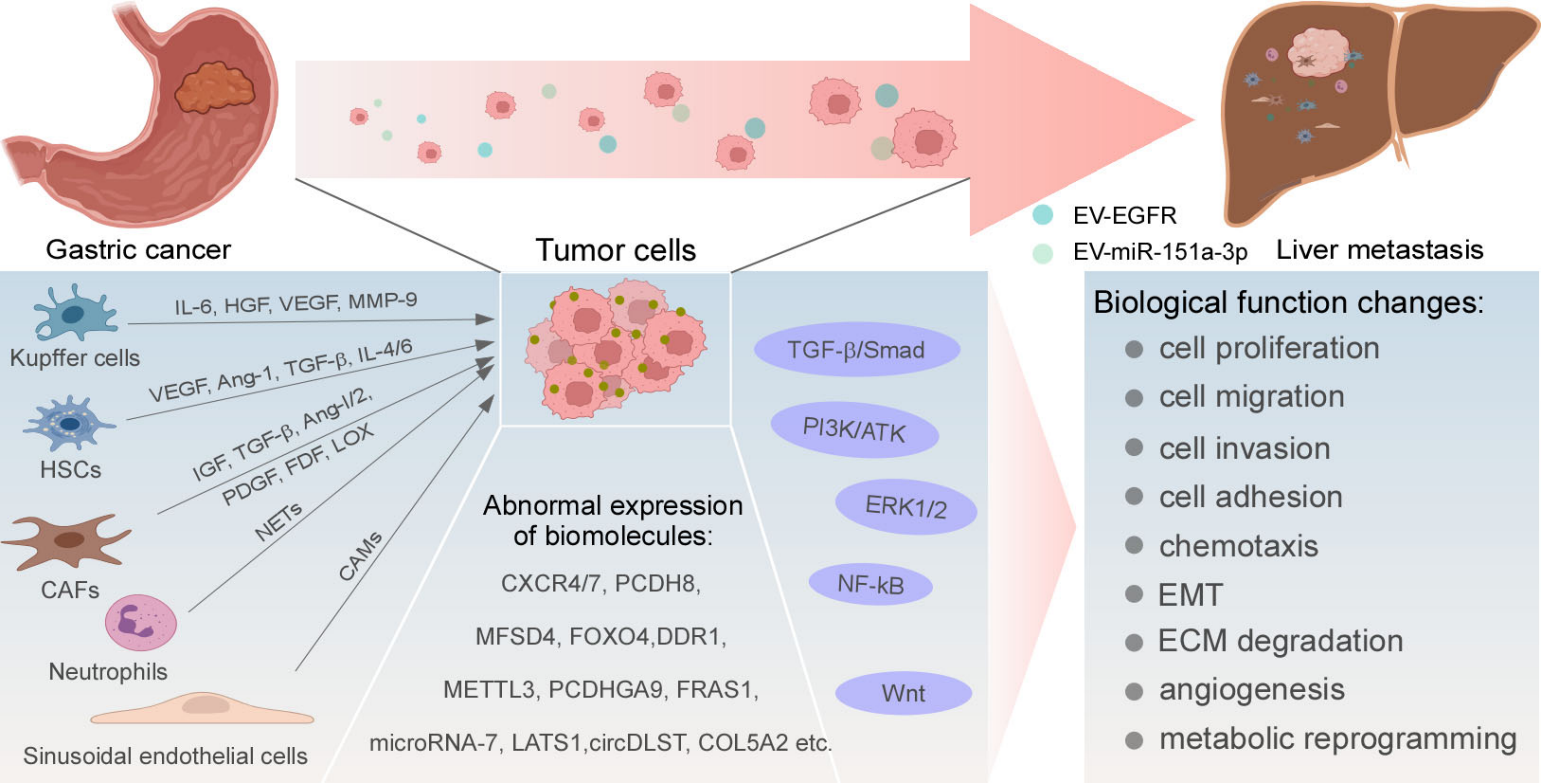
Regulatory mechanisms of GC liver metastasis. Abnormal expression and activation of biomolecules, signaling pathways, and cytokines produced by resident cells in liver TME drive uncommon alterations of GC cells in multiple cellular biological functions, ultimately leading to the development of liver metastasis.

Biomolecules are abnormally expressed in GC and mediate the occurrence of liver metastasis by altering various biological functions of tumor cells, such as chemotaxis, extracellular matrix (ECM) degradation, epithelial-mesenchymal transformation (EMT), metabolic reprogramming, and *TP53* mutations. The targeted inhibition of these molecules may achieve the purpose of inhibiting the occurrence and progression of liver metastasis (**Figure 1**).

### Chemotaxis

CXC motif chemokine receptor (CXCR) is a G-protein-coupled receptor, engaging in cell recruitment and chemotaxis. Multiple findings suggest that CXCR is highly expressed in GC and associated with growth and metastasis in various tumor models (22). Stromal cell-derived factor (SDF), also known as CXC motif ligand 12 (CXCL12), is the ligand of CXCR and is expressed in many organs, including the liver (23). Tumor cells expressing CXCR tend to be recruited to the place where their ligand SDF-1 is expressed (24). CXCL12 was increased in GC liver metastasis compared with normal liver tissue, the formation of liver metastasis was reduced when the expression of CXCR4 and CXCR7 was suppressed in GC by a mechanism that may inhibit the CXCR4/7-CXCL12 axis (25, 26). Additionally, Yu Zhang et al. found that the expression of DC-SIGN-related protein (DC-SIGNR) inhibited heterogeneous nuclear ribonucleoprotein K pseudogene 2 (HNRNPKP2), thereby enhancing the expression of CXCR4 and the downstream target gene of HNRNPKP2, finally promoting the selectivity of liver metastasis (27).

### ECM degradation

ECM is vital to maintain normal cellular homeostasis, which includes collagen, elastin, laminin, fibronectin, proteoglycan, and other matrix components (28). Matrix metalloproteinases (MMPs) can change the structure of ECM by degrading, remodeling, and hardening the matrix components, fiber proliferation, etc., which are closely related to tumor invasion and metastasis (29). Ying Lin et al. have believed that laminin subunit γ2 (LAMC2) encoding adherent laminin, significantly increased the number of liver metastatic tumors when protocadherin-8 (PCDH8) was overexpressed in GC cells. Mechanically, PCDH8 induced the interactions between ECM and cell receptors to promote the adhesion of GC cells in the liver parenchyma, leading to the development of liver metastasis (30). Analogously, Gui Ren et al. and Mitsuro Kanda et al. have observed that MMPs were involved in the structural changes of ECM and promoted the occurrence of GC liver metastasis. The former author has suggested that a high expression level of Coronin 3 was positively correlated with MMP-9 in GC cells, and the latter has thought that the expression of the major facilitator superfamily domain containing 4 (MFSD4) indirectly promoted MMP-2 expression. Both have believed that the degradation of ECM components by MMPs increased cell invasion *in vitro* and liver metastasis *in vivo* (31, 32). Meanwhile, Xionghu Shen et al. have considered that the occurrence of GC liver metastasis was related to the low expression of E-cadherin in ECM, which increased the invasion and metastasis ability of GC cells (33).

### EMT

EMT is an evolutionarily highly conserved developmental program, and associated with tumor metastasis, which imparts metastatic properties to tumor cells by increasing motility, invasiveness, cell dryness, and apoptotic resistance (34). It is often accompanied by the loss of apical-basal polarity, the disassembly of the epithelial cell-cell contacts, and the reorganization of the actin cytoskeleton structure (35). Studies have shown that the presence of EMT in GC liver metastasis. For example, Linna Su et al. have found that down-regulation of Forkhead Box O4 (FOXO4) promoted the expression of vimentin in GC mesenchymal cells, while that of E-cadherin did not significantly change (36). Mitsuro Kanda et al. believed that the expression of MFSD4 was positively correlated with the expression of EMT inhibitor occludin (OCLN), and high expression of MFSD4 inhibited EMT *in vitro* and liver metastasis *in vivo* (32). Discoidin domain receptor 1 (DDR1) was highly expressed in GC tissues and cell lines compared to adjacent tissues and normal cell lines. There was a statistically significant correlation between the expression of DDR1 and liver metastasis. Mechanistically, DDR1 overexpression led to low expression of E-cadherin and high expression of vimentin and snail, suggesting that DDR1 promoted GC liver metastasis *via* EMT (37).

### Metabolic reprogramming

Metabolic reprogramming is considered as a symbol of tumor progression. Nutrient acquisition and metabolic pathways can be reprogrammed by tumor cells to meet their bioenergy, biosynthesis, and redox requirements. One of the classic examples is the Warburg effect, in which malignant tumor cells use glycolysis to obtain a flux to meet the metabolic needs of proliferating cells regardless of the presence or absence of oxygen. Meanwhile, the oxidative phosphorylation pathway is inhibited (38). This process of tumor cells is also believed to participate in GC liver metastasis. Qiang Wang et al. have found that Methyltransferase-Like Protein 3 (METTL3) was significantly upregulated in GC tissues. Additionally, it enhanced the expression of glucose transporter 4 (GLUT4) and enolase-2 (ENO2), increasing glycolysis flux, and promoting liver metastasis (39). Furthermore, Qing Li et al. have found that CAFs-derived lysyl oxidase (LOX) facilitated the Warburg effect of tumor cells and increased the flux of glycolysis (40).

### *TP53* mutations

As a tumor suppressor gene, *TP53* is widely distributed in various tissues and has greatly contributed to the protection from developing cancer. Numerous studies have found that *TP53* gene is highly susceptible to mutations and associated with various diseases, including tumorigenesis and tumor metastasis. The metastasis-related mechanisms of *TP53* mutations mainly involve EMT, ECM interactions, activation of receptor tyrosine kinase (RYK) signaling, etc. (41). *TP53* mutations are also related to GC metastasis. Jieyun Zhang et al. have performed whole-exome sequencing of tumor and normal tissues from GC patients and found that *TP53* mutations were early drivers of metastasis and significantly associated with poor metastasis-free survival, but the relationship between *TP53* mutations and GC liver metastasis was not clear (42). However, Naoki Ikari et al. have found the correlation between *TP53* mutations and GC liver metastasis by next-generation sequencing, and the rate of *TP53* mutations was significantly higher in advanced GC patients with liver metastasis compared to those without liver metastasis (86.5% vs. 40.5%), although the exact mechanism of *TP53* mutations on liver metastasis needs to be further investigated (43).

## Signaling pathways mediate the metastasis spread of GC cells to the liver

Certain signaling pathways are involved in the transduction of extracellular molecular signals to intracellular signals, exerting certain biological effects. In recent years, it has been confirmed that multiple signaling pathways are linked to the occurrence of GC liver metastasis, such as Wnt, nuclear factor kappaB (NF-κB), extracellular regulated kinase 1/2 (ERK1/2), transforming growth factor-β (TGF-β)/Smad, PI3K/AKT, Hippo-YAP pathway, etc. (**Figure 1**).

### Wnt signaling pathway

Abnormal activation of Wnt signaling promotes tumor progression, which can be divided into classical Wnt/β-catenin, non-classical Wnt/Ca^2+^, and Wnt/PCP (44). In GC, the upregulation of tumor suppressor gene protocadherin gamma subfamily A9 (PCDHGA9) blocked TCF/LEF transcription by restraining the nuclear translocation of β -catenin, inhibiting liver metastasis (45). MIR17HG is a class of pre-miRNAs located on human chromosome 13, which can be spliced to produce six miRNAs, including miR-18a and miR-19a. In a study, miR-18a and miR-19a directly inhibited the expression of SMAD2 and activated the Wnt/β-catenin signaling pathway to mediate liver metastasis *in vivo*, whereas metastasis was suppressed when the MIR17HG-miR-18a/miR-19a signaling axis was inhibited (46). Non-classical Wnt signaling is also deemed to be engaged in the progression of liver metastasis. RYK, a receptor of the non-classical Wnt ligand Wnt5a, was highly expressed in GC tissues, promoting cell migration, invasion, and EMT *in vitro* and liver metastasis *in vivo* (47).

### NF-κB signaling pathway

NF-κB is a protein complex controlling DNA transcription. Numerous studies have shown that NF-κB participates in key steps of tumorigenesis and tumor metastasis. The NF-κB activity is usually influenced by the activation of multiple transcription factors, regulating the expression of its downstream target genes (48, 49). MicroRNA-7 mechanistically reduced the expression of p65 and p-p65-ser536, inhibiting NF-κB transcriptional activity and the expression of its downstream metastasis-related molecules vimentin, intercellular cell adhesion molecule-1 (ICAM-1), vascular cell adhesion molecule-1 (VCAM-1), vascular endothelial growth factor (VEGF), MMP-2, and MMP-9. Ultimately liver metastasis was suppressed (50). MicroRNA-10b has a similar regulatory role as microRNA-7 in GC. Expression of microRNA-10b promoted ectopic tumor growth and metastasis of tumor cells to the liver. Unlike microRNA-7, microRNA-10b increased the malignant phenotype of tumor cells by suppressing the expression of CUB and sushi multiple domains protein 1 (CSMD1) and up-regulating the expression of c-MYC, cyclin D1, and EMT markers (51). The tumor suppressor gene phosphatase and tensin homolog (PTEN) had an essential impact on inhibiting tumor growth and metastasis. Overexpression of PTEN inhibited cell proliferation and invasion *in vitro* and liver metastasis *in vivo*. The results might be due to the inhibition of the PI3K/NF-κB signaling pathway by PTEN, inhibiting the DNA binding of NF-κB on the FAK promoter and reducing the invasion of GC cells (52).

### ERK1/2 signaling pathway

ERK1/2 are the members of the mitogen-activated protein kinase family, which are involved in many cellular events, including cell proliferation, growth, differentiation, migration, survival, metabolism, and transcription. ERK1/2 are primarily phosphorylated in the cytoplasm and translocated to the nucleus and then regulate the transcription of related target genes (53). Abnormal activation of this signaling transduction pathway leads to the occurrence of metastasis. Jing Zhang et al. have found that expression of circDLST was dramatically elevated in GC tissues than in adjacent tissues. Its expression was positively correlated with cell viability, invasion *in vitro* and liver metastasis *in vivo*. It might have been caused by abnormal activation of the NRAS/MEK1/ERK1/2 signaling pathway and increased expression of proliferating cell nuclear antigen (PCNA) and MMP-2 (54). Higher expression of ubiquitin C-terminal hydrolase-L1 (UCHL1) in liver metastasis than in primary tissue could activate the ERK1/2 signaling, thus promoting the proliferation, migration, and invasion of GC cells (55).

### The roles of TGF-β, PI3K/AKT, and Hippo-YAP pathway

TGF-β, PI3K/AKT, and Hippo-YAP are related to GC liver metastasis, and dysregulation of these signaling pathways results in cell proliferation and invasion. Collagen type V alpha 2 chain (COL5A2) promoted GC liver metastasis, which might be caused by activating the TGF-β pathway (56, 57). High expression of extracellular matrix complex subunit 1 (FRAS1) activated the PI3K/AKT signaling pathway, which increased GC cell stemness, invasion, migration, responsiveness to oxygen stress, and liver metastasis. By the way, the cumulative liver recurrence rate was significantly increased in GC patients with high expression levels of FRAS1 (58). Moreover, ectopic expression of large tumor suppressor factor 1 (LATS1) inhibited GC cell proliferation and invasion *in vitro* and liver metastasis *in vivo*, whereas the depletion of LATS1 expression restored the invasive phenotype. The YAP pathway was required for LATS1-induced inhibition of cell growth and invasion. LATS1 restrained nuclear translocation of YAP and downregulated the expression levels of YAP, MMP 2, MMP 9, PCNA, and connective tissue growth factor (CTGF) (59).

## Liver resident cells provide favorable survival conditions for tumor cells to grow

Liver resident cells such as macrophages, neutrophils, hepatic stellate cells (HSCs), natural killer (NK) cells, hepatic sinusoidal endothelial cells, and fibroblasts constitute an important portion of the liver TME, which interacts with tumor cells by secreting growth factors to form a favorable growth microenvironment for tumor cells to survive in the liver (**Figure 1**).

### Kupffer cells (KCs)

KCs are special macrophages located in the liver, which can be shifted between M1 and M2 polar types, thus producing a bidirectional effect on tumor cells through different mechanisms. At the initial stage of tumor cell invasion, KCs can adhere to tumor cells, phagocytose them, or induce apoptosis. KCs also recruit NK cells to remove tumor cells together (60, 61). At the later stage, tumor cells escaping the killing effect of KCs can further utilize various growth factors such as interleukin- 6 (IL-6), hepatocyte growth factor (HGF), VEGF, and MMP-9 secreted by M2-type KCs to form an environment suitable for tumor cells to proliferate; thus liver metastasis is formed (62). Additionally, there are carcinoembryonic antigen (CEA) receptors on the surface of KCs. They can release inflammatory cytokines, when activated. Then the expression of cell adhesion molecules (CAMs) is increased and the production of NO is reduced, forming a conducive environment for tumor cells to survive (63).

Small extracellular vesicles (sEVs) participate in intercellular communication transduction. Exosomes containing miR-151a-3p secreted by GC cells are highly expressed in the plasma of GC patients with liver metastasis and predict poor prognosis. sEV-miR-151a-3p can be absorbed by KCs and reduce the transcriptional activity of SP3 by inhibiting SUMO1 translation in an N6-adenosine-dependent manner. These alterations promote TGF-β1 trans-activation in KCs, activate the SMAD2/3 pathway and subsequently enhance stem-cell-like properties of GC cells. Meanwhile, in mouse models, sEVs containing miR-151a-3p promote liver metastasis (64).

### HSCs

Under normal conditions, HSCs are inactive and do not express α-smooth muscle actin (α-SMA). When tumor cells invade the liver parenchyma, HSCs are stimulated to acquire a myofibroblast-like phenotype and produce type I and IV collagen, promoting the formation of ECM and leading to liver fibrosis. The aggressiveness of tumor cells is also enhanced (61). Inhibiting the activation of HSCs can attenuate HSCs-induced tumor growth and angiogenesis (65). On the other hand, HSCs can produce various cytokines, such as VEGF, angiopoietin-1 (Ang-1), TGF-β, HGF, IL-4, IL-6, and other cytokines, leading to the occurrence of liver metastasis by promoting the formation of pre-metastasis microenvironment (62, 66–68).

Recent evidence has demonstrated that EVs secreted by GC cells contain a high amount of epidermal growth factor receptor (EGFR), and expression of HGF is increased in liver metastasis. After the co-culture with primary mouse liver cells, EGFR-rich EVs translocated to the liver could inhibit miR-26a/b and promote the expression of HGF in liver cells, while high expression of HGF could interact with the receptor of HGF on GC cells, thus promoting the colonization and growth of GC cells in the liver. Further fluorescence localization revealed that EGFR-rich EVs from GC cells could be absorbed by HSCs. HSCs might absorb EGFR-rich EVs secreted by GC cells, thus mediating a series of processes in liver metastasis (69). However, further study is required to investigate whether only HSCs in primary liver cells had this effect or multiple liver resident cells mediated this process together.

### CAFs

In response to persistent liver injury or stimulation from tumor cells, HSCs are activated and become converted to CAFs, and normal fibroblasts can also be transformed into CAFs (70). CAFs can secrete growth factors including insulin like growth factor (IGF), IL-6, and TGF-β, which stimulate the proliferation and invasion of malignant tumor cells through paracrine signaling in TME (71). Additionally, under hypoxic conditions, CAFs activate the AKT signaling, secrete vascular growth factors, including EGF, platelet-derived growth factor (PDGF), fibroblast growth factor (FDF), Ang-1, and Ang-2, which promote the formation of blood vessels, and provide growth conditions for proliferation of tumor cells in the liver (72). In GC liver metastasis, the expression of LOX in CAFs was up-regulated, which promoted the proliferation of tumor cells and suggested a poor prognosis. CAFs expressed more LOX when GC cells migrated into the liver and secreted TGF-β1, enhancing the Warburg effect mediated by the AKT-P70S6K-HIF1-α pathway to promote GC cells proliferation and liver metastasis (40).

### Neutrophils, NK cells, hepatic sinusoidal endothelial cells, and liver parenchymal cells

Normally, neutrophils can directly or indirectly remove tumor cells when tumor cells invade tissues. However, recent studies have found that neutrophils can be mobilized to the liver by driving factors S100A8 and S100A9, forming neutrophil extracellular traps (NETs), resulting in the retention of tumor cells in the “traps”, further promoting the adhesion, proliferation, and invasion of tumor cells and the formation of liver metastasis (73, 74). NK cells are important for innate immune response and produce cytotoxic substances that effectively defend against external pathogens (75). Changes in the liver TME, such as hypoxia, metabolites of tumor cells, acidic conditions, cytokines, and growth factors (IL-10 and TGF-β) can inhibit the killing effect of NK cells (76). In colon cancer, lactic acid mediates liver microenvironment acidification, induces NK cell apoptosis, and promotes liver metastasis (77). Hepatic sinusoidal endothelial cells express CAMs that can increase the adhesion of tumor cells and help tumor cells migrate to the space of Disse, where they escape the killing effect of KCs and NK cells (78). EMT is also induced by hepatic sinusoidal endothelial cells (63, 79). Additionally, the contact adhesion between liver parenchymal cells and tumor cells is considered to be one of the earliest events in the formation of liver metastasis, which can secrete IGF-1 to promote the liver colonization of lung cancer cells through paracrine mechanisms (80). However, the cognition of these resident cells in the progression of GC liver metastasis is still limited, and the related mechanisms need to be further studied.

## Potential therapeutic targets may inhibit the progression of GC liver metastasis

**Figure 2:**
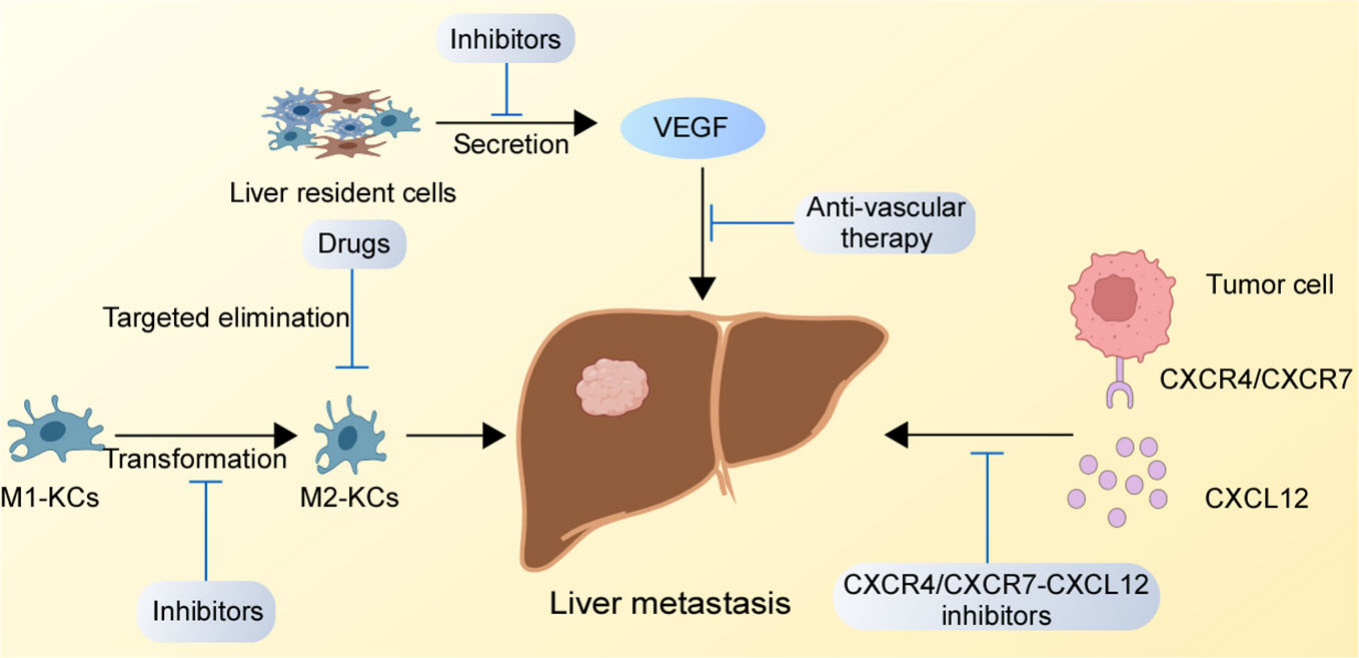
Potential therapeutic targets for GC liver metastasis. The inhibition of the CXCR4/CXCR7-CXCL12 axis in the liver, suppression of M1-type KCs to M2-type KCs conversion, and blockade the binding of receptors secreted by liver resident cells binding to receptors on GC cells might destroy the TME of liver metastasis.

The liver is one of the essential organs in our body, and the occurrence of liver metastasis is a serious threat to a human organism. Conventional surgery and drugs cannot completely eradicate liver metastasis. Therefore, the development of reasonable and effective targeted therapies for liver metastasis has become urgent. Through an in-depth understanding of the regulatory mechanisms of GC liver metastasis, we found potential therapeutic targets that might have therapeutic effects on the suppression of GC liver metastasis, which are presented in **Figure 2**.

### Targeting the CXCR4/CXCR7-CXCL12 chemokine axis

Studies have shown that the CXCR4/CXCR7-CXCL12 axis mediates the selective metastasis of GC cells to the liver. Similar mechanisms have been observed in liver metastasis of cholangiocarcinoma and colon cancer (81, 82), and CXCR4 antagonist AMD300 has effectively inhibited the progression of liver metastasis in animal experiments (83). Thus, we speculate that the inhibition of the CXCR4/CXCR7-CXCL12 chemokine axis by drug targeting might have a blocking effect on GC liver metastasis. Additionally, several drugs targeting this mechanism have been applied in clinical trials. In a phase 1/2 clinical study about relapsed or refractory acute myeloid leukemia (NCT00512252), plerixafor has shown encouraging results in objective disease response rates by antagonizing the CXCR4/CXCL12 axis to promote chemotherapy sensitivity (84). Plerixafor has also been used in clinical trials of recurrent high-grade glioma. Being combined with bevacizumab, plerixafor has been well tolerated and significantly inhibited biomarkers of anti-vascular therapy, such as VEGF and Ang-2. However, the median overall survival and progression-free survival have not improved significantly due to limited clinical cases, which needs to be further observed and discussed (85). Injection site reactions and adverse gastrointestinal symptoms (abdominal pain, diarrhea, nausea and vomiting) are some of the adverse effects that are likely to occur due to plerixafor (86). Additionally, other chemokine axis inhibitors, such as MSX-122, USL311 and Olaptesed pegol are still in clinical trials and their efficacy needs to be verified (87). This is a significant reason to believe that those drugs might become a viable option in the future.

### Targeted KCs

Targeted therapy against KCs might be a promising therapeutic strategy in the early stage of liver metastasis. On the one hand, the killing effect of KCs on tumor cells can be enhanced by converting M2-type into M1-type KCs. On the other hand, M2-type KCs can be depleted by drugs, thus inhibiting the formation of a pre-metastatic ecological niche suitable for growth and reducing the colonization of tumor cells (88). Due to the dual action of KCs on tumor cells, the development of drugs with clear effects is complicated. Some questions need to be further clarified, such as whether the consumption of KCs will also target the toxic M1 KCs, but it is still a prospective approach.

### Targeting the formation of metastatic focal vessels

From the mechanisms of the liver microenvironment, we found that different liver resident cells such as KCs and HSCs, can secrete vascular growth factors such as VEGF to promote the early vascularization of tumor cells in the liver. Therefore, whether targeting the metastatic focal vessels will reshape TME and create an ecological niche unfavorable for tumor cells to grow is a question requiring further investigation. Drugs targeting tumor blood vessels such as bevacizumab have been used and have produced a positive effect in clinical trials of liver metastasis of colon cancer by combining with conventional chemotherapy drugs (89). This might become an alternative treatment option for patients with GC liver metastasis.

## Discussion and outlook

In this review, we summarized biologically relevant studies on GC liver metastasis, including the aberrant expression of biomolecules in GC cells, the abnormal transduction of relevant signaling pathways, and the interaction between resident cells and GC cells in the liver microenvironment, leading to the formation of pre-metastatic ecological niches, which promotes the ability of tumor cells to proliferate, invade, and migrate. Generally, liver metastasis is a complex process. Although a lot of studies have investigated the involved molecular mechanisms, there are still many questions that deserved further exploration, such as whether GC has a special mechanism for liver metastasis selectivity different from other distant organ metastasis (90). If this mechanism can be clarified, it is believed that there will be a new idea for its prevention, diagnosis, and treatment. Additionally, whether liver metastasis from other tumors, such as colorectal cancer, pancreatic cancer, and lung cancer, are mediated by similar mechanisms observed in GC, the blockade of the same metastasis mechanism might have similar therapeutic effects on liver metastasis from these organs (91). This could be an interesting topic for future research.

For inoperable GC patients, including those with liver metastasis, chemotherapy has shown positive effects on tumor suppression, but the complexity of tumor cells leads to chemotherapy resistance and disease progression in many patients, which is an urgent problem to overcome for patients with advanced GC. The advent of targeted therapies and immunotherapy offers effective solutions for these patients. For example, anti-vascular therapies with ramucirumab targeting VEGFR-2, either as monotherapy or in combination with paclitaxel, have benefited survival (92, 93). Compared to chemotherapy alone, the median survival with trastuzumab in combination with conventional chemotherapy has improved in HER-2-positive advanced GC patients (94). Immunotherapy, such as pembrolizumab and nivolumab, in combination with chemotherapy has also shown promising results in advanced GC (95, 96). However, individual heterogeneity and differences in metastasis location result in different disease responses to the same targeted therapy and immunotherapy.

Research has revealed that changes in TME can have an ignorable role in GC metastasis. The occurrence of liver metastasis is inseparable from the interaction between GC cells and liver resident cells. KCs, HSCs, and CAFs all have specific behaviors leading to the colonization of GC cells in the liver (62, 65, 72). The elucidation of these mechanisms might provide specific therapeutic targets for GC liver metastasis, such as the inhibition of the shift from M1 to M2 type KCs, inhibition of the CXCR4/CXCR7-CXCL12 axis between liver and GC cells, and inhibition of the secretion of cytokines, such as VEGF, by liver resident cells. Additionally, abnormal molecules such as PCDH8, FOXO4, etc., contribute to the development of GC liver metastasis through ECM degradation, EMT and other behaviors (30, 36). The feasibility of combination therapy provides us with new targeted therapeutic options, such as combining anti-PD-1/PD-L1 or trastuzumab regimens for HER-2-positive GC patients with these specific therapeutic targets for liver metastasis, which might have promising results for the treatment. More clinical data are needed to verify the rationale for these novel treatment options.

Overall, the treatment of advanced GC will be a serious issue in developing countries for the next 10-20 years. Although new treatment options are now being further evaluated in the clinic, such as targeting claudin 18.2, which is considered as a new target of advanced GC and cytotoxic T-lymphocyte antigen 4 (CTLA-4) immunotherapy (97, 98), very few of these options offer substantial survival benefits. However, there are no clinical trials with specific targets for GC liver metastasis. This article reviewed the latest developments in the regulatory mechanisms of GC liver metastasis, hoping to identify effective therapeutic targets to improve patients’ survival.

## Author contributions

DL performed the literature search and wrote the manuscript. XZ and LJ presented the ideas for the article and revised the manuscript. All authors contributed equally to this article and approved the submitted version.

## Funding

This work was supported by the open research funds from the Sixth Affiliated Hospital of Guangzhou Medical University, Qingyuan People’s Hospital (202011-202), the Natural Science Foundation of China (82273464, 81972619, 81672874), the Natural Science Foundation of Guangdong Province (2021A1515012477, 2022A1515012260), the Basic and Applied Research Projects of Guangzhou Science and Technology Bureau (202002030067), the Key Discipline of Guangzhou Education Bureau (Basic Medicine) (201851839), the Natural Science Foundation research team of Guangdong Province (2018B030312001), the Innovative Academic Team of Guangzhou Education System (1201610014).

## Conflict of interest

The authors declare that the research was conducted in the absence of any commercial or financial relationships that could be construed as a potential conflict of interest.

## Publisher’s note

All claims expressed in this article are solely those of the authors and do not necessarily represent those of their affiliated organizations, or those of the publisher, the editors and the reviewers. Any product that may be evaluated in this article, or claim that may be made by its manufacturer, is not guaranteed or endorsed by the publisher.

## Glossary

**Table:**

GC	gastric cancer
TME	tumor microenvironment
MDSC	myeloid-derived suppressor cell
TAM	tumor-associated macrophage
CAF	carcinoma-associated fibroblast
ECM	extracellular matrix
EMT	epithelial mesenchymal transformation
CXCR	CXC motif chemokine receptor
SDF	stromal cell-derived factor
CXCL12	CXC motif ligand 12
DC-SIGNR	DC-SIGN-related protein
HNRNPKP2	heterogeneous nuclear ribonucleoprotein K pseudogene 2
MMP	matrix metalloproteinase
LAMC2	laminin subunit γ2
PCDH8	protocadherin-8
MFSD4	major facilitator superfamily domain containing 4
FOXO4	forkhead box o4
OCLN	occludin
DDR1	discoidin domain receptor 1
3'UTR	3’ untranslated region
METTL3	methyltransferase-like protein 3
GLUT4	glucose transporter 4
ENO2	enolase-2
LOX	lysyl oxidase
NF-κB	nuclear factor-kappaB
ERK	extracellular regulated kinase
TGF-β	transforming growth factor-β
PCDHGA9	protocadherin gamma subfamily A9
RYK	receptor-like tyrosine kinase
ICAM-1	intercellular cell adhesion molecule-1
VCAM-1	vascular cell adhesion molecule-1
VEGF	vascular endothelial growth factor
CSMD1	CUB and sushi multiple domains protein 1
PTEN	phosphatase and tensin homolog
PCNA	proliferating cell nuclear antigen
UCHL1	ubiquitin C-terminal hydrolase-L 1
COL5A2	collagen type V alpha 2 chain
FRAS1	fraser extracellular matrix complex subunit 1
LATS1	large tumor suppressor factor 1
CTGF	connective tissue growth factor
HSC	hepatic stellate cell
NK	natural killer
KC	kupffer cell
HGF	hepatocyte growth factor
CEA	carcinoembryonic antigen
CAM	cell adhesion molecule
EV	extracellular vesicle
α-SMA	α-smooth muscle actin
Ang-1	angiopoietin-1
EGFR	epidermal growth factor receptor
IGF	insulin like growth factor
PDGF	platelet-derived growth factor
FGF	fibroblast growth factor
NET	neutrophil extracellular trap
CTLA-4	cytotoxic T-lymphocyte antigen 4
